# A Comparative Study between Peptic Ulcer Perforation Score, Mannheim Peritonitis Index, ASA Score, and Jabalpur Score in Predicting the Mortality in Perforated Peptic Ulcers

**DOI:** 10.1055/s-0042-1743526

**Published:** 2022-08-02

**Authors:** Aboli Koranne, K G. Byakodi, Vasant Teggimani, Vijay V. Kamat, Abhijith Hiregoudar

**Affiliations:** 1 Department of Surgery, Karnataka Institute of Medical Sciences, Hubli, Karnataka, India

**Keywords:** perforated peptic ulcer, mortality, peptic ulcer perforation score, Mannheim peritonitis index, Jabalpur score, American Society of Anesthesiologists score

## Abstract

**Introduction** Peptic ulcer disease continues to be a major public health in most developing countries despite the advances in medical management. The incidence of perforations remains high and has the highest mortality rate of any complication of ulcer disease. Risk stratification of cases will lead to better preoperative management and efficient utilization of intensive care unit resources. The purpose of the present study is to compare different existing scoring systems and identify the most accurate predictor of mortality in perforated peptic ulcer (PPU) cases.

**Materials and Methods** This is an observational study conducted in Karnataka Institute of Medical Sciences, Hubli, India. All cases of PPU disease admitted from December 2017 to August 2019 who were treated surgically were included in the study. Demographic data were collected and peptic ulcer perforation (PULP) score, Mannheim peritonitis index (MPI), American Society of Anesthesiologists (ASA) score, and Jabalpur score (JS) were calculated for individual patient and compared. The patient was followed up during the postoperative period.

**Observation** A total of 45 patients were included in the study with a mean age of 42.5 years. Most of the patients presented with 24 hours of the onset of symptoms. Nonsteroidal anti-inflammatory drug use was noted in 8.9% patients, and steroid use was present in 2.2% patients. Of the 45 patients, 7 deaths were reported. Between the various scoring systems, the MPI and JS were better predictors of mortality with a *p* -value of <0.001 and 0.007, respectively. In contrast, the PULP and ASA scores had *p* -value not statistically significant. However, the PULP score was a better predictor of postoperative complication with a *p* -value of 0.047.

**Conclusion** Of the four scoring systems validated, the MPI and JS were better predictors of mortality in the given population. PULP score is a better predictor of postoperative complications in the present study.

Peptic ulcers are defined as erosions in the gastric or duodenal mucosa that extend through the muscularis mucosae. 1 This includes duodenal, gastric, and marginal ulcers. It continues to be a major public health problem throughout the world, more so in the developing nations. Although the mortality rates have declined in the past few decades, mortality rates due to its complications such as perforation and hemorrhage remain high. The role of operative therapy is mainly in the emergency or semielective treatment of complications such as perforation, obstruction, and bleeding.

The decline in the overall incidence and surgical intervention in peptic ulcer disease is attributed to the increased knowledge of ulcer pathogenesis, particularly the role of *Helicobacter pylori* and the development of proton pump inhibitors. The incidence of complications of peptic ulcer disease is still around 20%. Perforations have the highest mortality rate of any complication of ulcer disease approaching 15%. 1 About every fifth patient with ulcer perforation present with signs of sepsis. A careful preoperative assessment of the clinical severity of cases will help achieve an optimal outcome of disease.

Several studies have been conducted to identify potential risk factors of poor outcome and mortality. In most studies, old age, male population, nonsteroidal anti-inflammatory drug (NSAID) use, steroid use, and delayed presentation with surgery performed >24 hours after symptoms are the common predictors of mortality and morbidity. 2 3 4 5 In fact, a Danish study cohort reported that delay in surgery beyond 24 hours of presentation decreases the probability of survival by 2.4%. 6

The management of peptic ulcer perforations (PULPs) is mainly surgical. The recent World Society of Emergency Surgery (WSES) consensus recommends expectant nonoperative management for the most selected cases only. It also recommends utilization of scoring systems to predict the mortality in perforated peptic ulcer (PPU) cases. 7

Several scoring systems for outcome prediction have been reported, yet none appears to be superior, and most are investigated in isolation. Among the most frequently used are the American Society of Anesthesiologists (ASA) physical status classification system, the Boey score, and the more recently introduced PULP score. In the Indian population, the most widely used scores are the ASA and Boey scores. The PULP score, being recently developed, lacks validity. The Jabalpur score (JS) was formulated in India and hence higher efficacy may be expected due to demographic similarities. The present study is conducted to validate the PULP score on the Indian population and to compare the efficacy of the four scores.

## Objectives

- To study the efficacy of PULP scores in predicting the mortality in PPU patients.
- To compare the PULP score with Mannheim peritonitis index (MPI), ASA score, and JS in predicting the mortality in PPU patients.

## Methodology

This is a prospective observational study. The study is conducted on patients admitted to the Surgical Emergency Department of Karnataka Institute of Medical Sciences, Hubli, India. It is a city located in the northwestern part of the state of Karnataka. The catchment population is roughly ∼9 lakh.

All cases of acute abdomen presenting to casualty were subjected to erect X-ray of the abdomen to diagnose a perforation. Computed tomography (CT) scan was used only subject to availability. Preoperative investigations include blood counts, arterial blood gas, albumin levels, creatinine, HIV status, and chest X-ray. Routine ultrasound was not done. Specific history pertaining to comorbidities such as diabetes mellitus, hypertension, renal or cardiac abnormalities, and cirrhosis was documented specifically. Chronic use of NSAIDs or steroids was noted. All the cases were subjected to emergency open surgery. Laparoscopic surgery was not performed on any case due to nonavailability in the emergency setup. Any patient treated conservatively was excluded from the surgery. Intraoperatively, perforations other than duodenal or gastric were excluded as were cases with any underlying malignancy (confirmed by biopsy). Graham's omental patch repair was done in all cases with additional anterior gastrojejunostomy done in four of those cases. No definitive procedure was undertaken in any of the cases. Postoperatively, patients were monitored for duration of stay and development of any complications. There was no uniform antibiotic regimen and duration and type of antibiotic was personalized according to general condition and degree of contamination. Routine microbiological examination for contaminants was not done. No relaparotomy was done in any patient. Patients who were discharged were followed up after 2 weeks.

Definition of parameters:

1. Shock is defined as a blood pressure <100 mm Hg and a heart rate of >100 beats per minute.
2. Postoperative complications include respiratory infections, surgical site infection, intra-abdominal collections, and leak.
3. Mortality is defined as death occurring within 30 days of surgery.

### ASA Score

ASA score is a subjective assessment of the overall health status of a patient and is based on six classes (I–VI):

I—a normal healthy patient
II—a patient with mild systemic illness
III—a patient with severe systemic illness
IV—a patient with a severe systemic illness that is a constant threat to life
V—Moribund patient who is not expected to survive without the surgery
VI—a declared brain-dead patient whose organs are being removed for donor purposes.

### Mannheim Peritonitis Index

The MPI was developed based on a retrospective analysis of data in which 20 risk factors were considered. Of these, only eight were found to be significant and these were included in the final score. These are age, sex, duration of perforation, presence of malignancy, presence of organ failure, peritonitis, origin of sepsis, and nature of exudates. Scores were categorized by severity into mild, moderate, and severe with scores of <21, 21 to 29, and >29, respectively.

### PULP Score

PULP score was developed by Moller et al 8 to predict the 30-day mortality in PPUs. The parameters included are age, comorbid malignancy or acquired immunodeficiency syndrome (AIDS), presence of cirrhosis, steroid use, shock on admission, duration of perforation, serum creatinine, and ASA score. Patients are divided into low-risk patients, with less than 25% risk of mortality (a score of <7 points), and high-risk patients, with more than 25% risk of mortality (a score of > 7 points).

### Jabalpur Score

The parameters included are perforation to operation (P-O) interval, age, mean systemic blood pressure, heart rate, and serum creatinine. Total score is 21. Scores <9 were categorized as low risk and those >9 as high risk.

All the data were collected in a pro forma, and the scores were calculated individually for all the cases.

### Statistical Analysis

Various scoring systems (PULP, MPI, ASA, and JS) were cross-tabulated against mortality and morbidity (in terms of postoperative complications), and chi-square test was done to assess association between different scoring systems and death and postoperative complications.

The validity (sensitivity and specificity) of the scoring system was calculated using the existing predefined cutoffs from literature. The sensitivity and specificity were expressed as percentages. Youden's J statistics was computed as J = sensitivity + specificity − 1.

Receiver operating characteristic curve was plotted to identify suitable cutoffs to predict mortality from our data. The sensitivity and specificity for various cutoffs were reported, and best cutoff was chosen from the value with highest Youden's index.

All statistical tests were two sided, and *p* -values of less than 0.05 were considered statistically significant. Statistical analysis was done using the software Stata version 12.

## Results

**Fig. 1:**
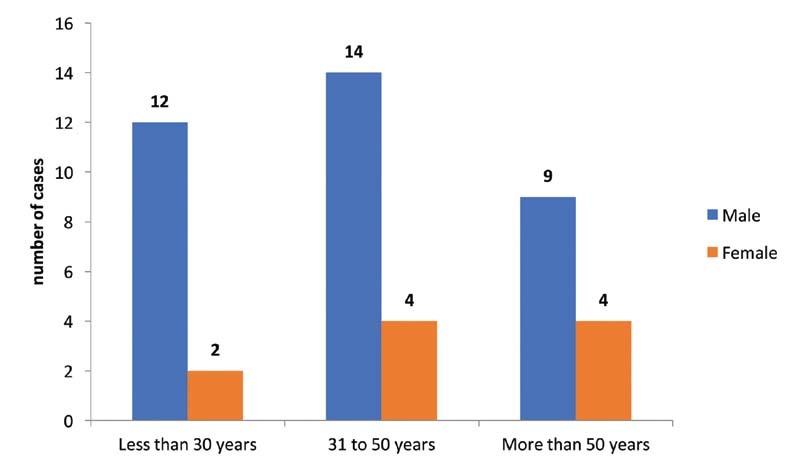
Age and gender distribution of participants.

**Fig. 2:**
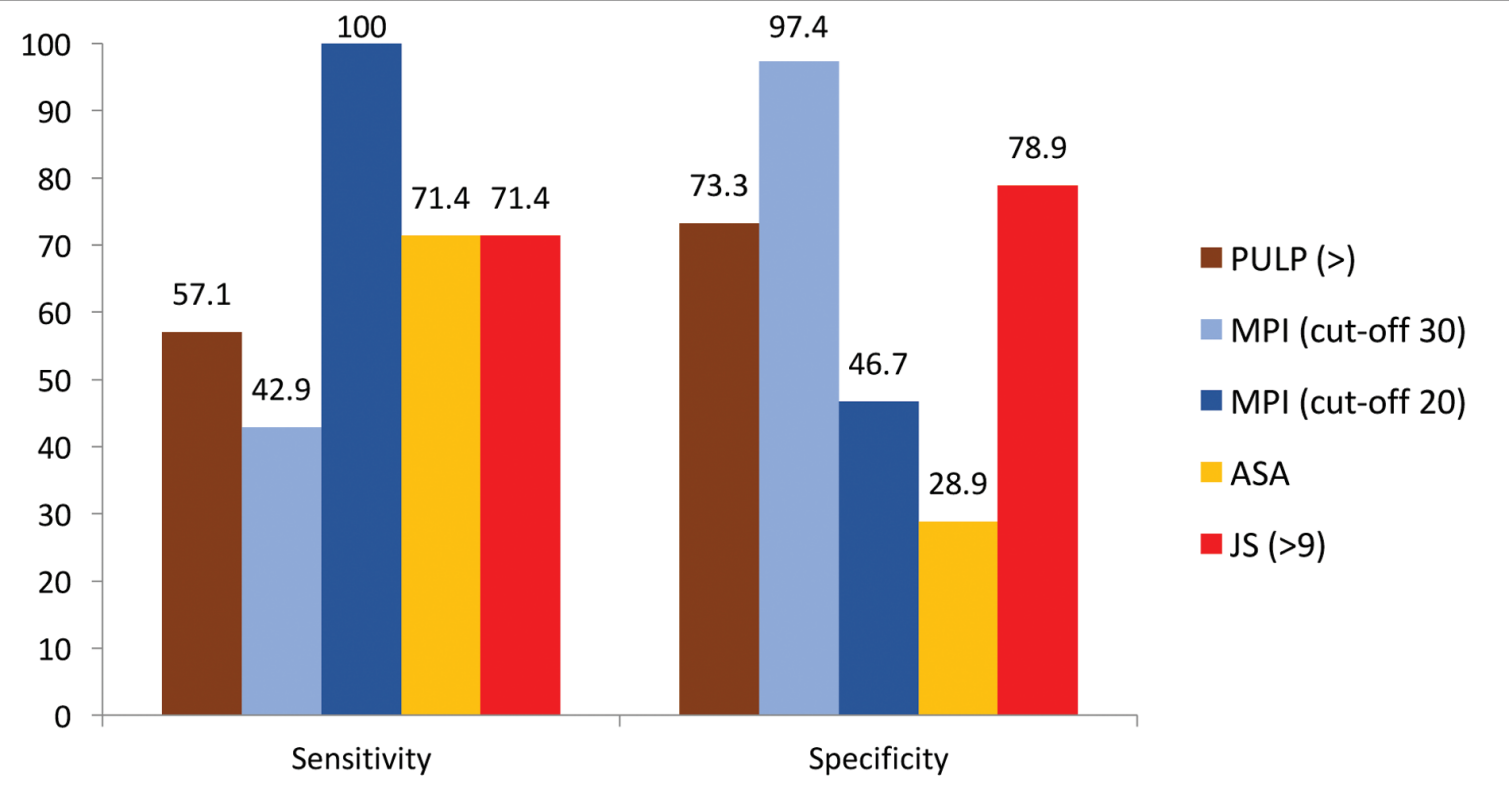
Comparison of sensitivity and specificity of various scoring systems—mortality. ASA, American Society of Anesthesiologists; JS, Jabalpur score; MPI, Mannheim peritonitis index; PULP, peptic ulcer perforation.

A total of 45 patients were included in the study from December 2017 to August 2019. The mean age of the population was 42.4 (range: 19–85) years, with 33 males and 10 females. Their distribution across age groups is shown in Fig. 1 . None of the patients had active AIDS, chronic liver disease, or malignancy. NSAID use was reported in 8.9% and steroid use in 2.2% of the patients. The average P-O interval as considered from onset of symptoms ranged from 1 to 5 days with the majority (42.2%) presenting on day 1 of symptoms. Shock was a presenting feature in 22.2% of the patients. Pneumoperitoneum on X-ray was detected in 100% of the patients. CT scan was not done routinely due to nonavailability. All patients underwent laparotomy on the same day. Graham's omental patch repair was done in all cases with anterior gastrojejunostomy done in four of those. No laparoscopic procedure was done in the emergency setup. Microbiological assessment of exudates was not done. All cases of gastric ulcer were subjected to biopsy to rule out malignancy. In the postoperative period, seven deaths occurred within 30 days of the procedure, all in the same admission. The predictive powers of the four scores were calculated using the chi-square test. Of these, the MPI and JS were found to be superior with a *p* -value of 0.001 and 0.007, respectively. A comparison of the sensitivity and specificity of individual scores is shown in Fig. 2 .

**Fig. 3:**
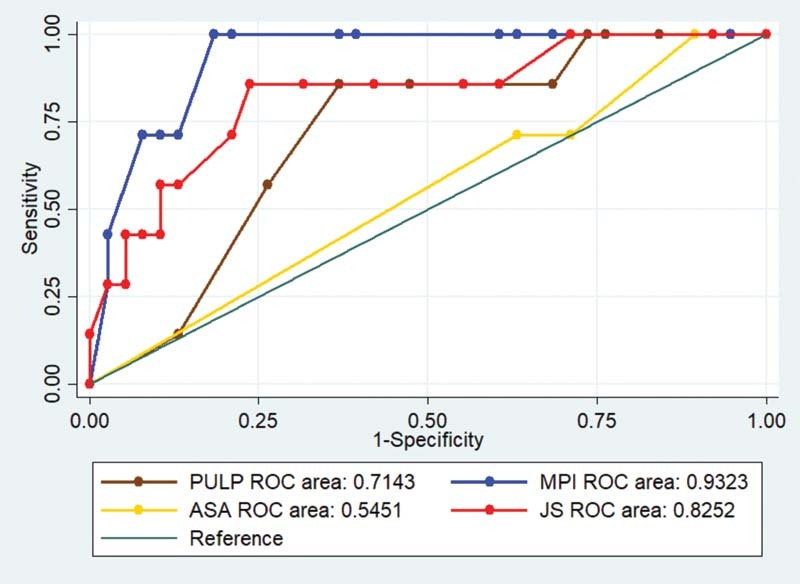
Comparison of ROC curve of all the scores. ASA, American Society of Anesthesiologists; JS, Jabalpur score; MPI, Mannheim peritonitis index; PULP, peptic ulcer perforation; ROC, receiver operating characteristic.

The average duration of stay was 10.5 days. Of the four scores, MPI was found to correlate the most with the duration of stay. Postoperative complications were noted in 4 out of 45 cases which was predicted accurately by the PULP score ( *p* = 0.047), while none of the other scores was statistically significant. The area under the curve (AUC) for the scores is shown in Fig. 3 .

## Discussion

The application of scoring systems is many folds. Although the positive predictive values of scores are varied and may not be uniform, they help assure a certain level of quality of care given, with efficacious allotment of resources to the critically ill. They are also an indirect tool of data collection and thus help in improvising the treatment protocol which is in a constant flux. While the search on an ideal score for PPUs is awaited, there are definite advantages in utilizing the existing models till then ( Table 1 ).

**Table 1:** Predictive capacity of various scoring system in predicting mortality in perforated peptic ulcer disease

	Died ( *n* = 7)	Not died ( *n* = 38)	Total ( *n* = 45)	*p* -Value
PULP score				
> 7	4 (57.1%)	10 (26.3%)	14 (31.1%)	0.105
≤7	3 (42.9%)	28 (73.7%)	31 (68.9%)	
MPI				
≥30	3 (42.9%)	1 (2.6%)	4 (8.9%)	<0.001 a
21–29	4 (57.1%)	13 (34.2%)	17 (37.8%)	
< 21	0	24 (63.2%)	24 (53.3%)	
ASA category				
Categories 3 and 4	5 (71.4%)	27 (71.1%)	32 (71.1%)	0.984
Categories 1 and 2	2 (28.6%)	11 (28.9%)	13 (28.9%)	
Jabalpur score				
≥9	5 (71.4%)	8 (21.1%)	13 (28.9%)	0.007 a
< 9	2 (28.6%)	30 (78.9%)	32 (71.1%)	

Abbreviations: ASA, American Society of Anesthesiologists; MPI, Mannheim peritonitis index; PULP, peptic ulcer perforation.

a Statistically significant *p* -value ≤0.05.

PULP is one of the most common causes of peritonitis accounting for up to 50 to 70% cases in some population-based studies. 9 10 While definitive surgery in the elective setting has declined, surgery is the mainstay of treatment for perforated ulcers. Several studies have been done to identify the common causes and factors influencing mortality, the most common being old age, male gender, smoking, delayed presentation, and size of the perforation. 2 3 4

While peptic ulcers may be seen equally in both genders, perforations were found to be more common in males as is reflected in the present study. This was attributed to smoking and alcohol consumption. In the geriatric population, NSAID and steroid were causative factors.

Several scoring systems have been studied to predict poor outcome in the critically ill and has been extended to predict outcome in PULPs. The updated WSES guidelines for the management of perforated ulcers have advocated the use of scoring systems to predict mortality. These along with scoring systems such as the sequential organ failure assessment help in focused aggressive resuscitation in susceptible individuals. In fact, a study conducted by Hoyt et al 11 have shown that using machine learning algorithm to predict sepsis can reduce mortality by 39.5% and duration of hospital stay by 32.3%. 12

In this regard, several attempts have been made to formulate a single effective scoring system. Most of the scoring systems currently in use are nonspecific, that is, they do not particularly include only PULPs. Only a handful of scoring systems focus on PULPs specifically. The scoring systems currently in use include the APACHE II, POMPP, ASA, Boey score, Charlson's comorbidity index, PULP score, MPI, and Jabalpur scoring system among others. Of these, only the PULP score and JS are specific for PULPs. In the Indian demographic, only a few studies have been conducted in this regard, although the Jabalpur scoring system was formulated on the Indian population. The PULP score remains to be validated in a large-scale study.

In the original study conducted by Moller et al, the PULP score was formulated on a cohort from Denmark and was compared with the ASA and Boey scores. The PULP score performed well with an AUC of 0.83. In the present study, the PULP score was found to be better predictor of mortality with an AUC of 0.7143. Though this is not statistically significant, the small sample size may factor in a bias.

Another study was conducted by Thorsen et al which compared the efficacy of PULP, Boey, and ASA scores. The results obtained found that the PULP and ASA scores performed equally well with an AUC of 0.79. 12 Another study was conducted by Anbalakan et al which compared PULP score, MPI, Boey score, and ASA score. In this study, the AUC was highest for MPI (0.77) followed by ASA and PULP score (AUC 0.75). The application of the PULP score was thought to be limited due to the demographic differences in the population as compared with the original study. 13 The present study also showed comparable results, with the MPI being superior to the PULP score in predicting the mortality.

In a study conducted by Prakash et al, the JS was compared with MPI to compare the efficacy in predicting mortality in PULP patients. This study conducted in a cohort of 150 patients found that the JS had a higher AUC (96%) than the MPI (95%). In a population like India, with limited resources, the JS may be superior. 14 The present study however showed that the MPI score had a higher AUC compared with the JS (0.93 vs. 0.82). The JS also was a good predictor of mortality with a significant *p* -value of 0.007.

A study was conducted by Menekse et al to formulate a new scoring system and was compared with the existing ASA, Boey, and PULP scores. The PULP score had an AUC of 0.955 and the ASA score had an AUC of 0.914. 15 However, it was found that the PULP score was not easy to use and the definition of time interval was modified as from perforation onset to surgery. For this reason, the prediction of PULP may have been higher in this study than previously reported ones. The PULP score was compared with the JS in another study conducted by Sundararajan. It was found that although neither of the two scores was ideal in predicting the mortality, the PULP score fared better at a cutoff of 4.5 with a sensitivity of 90% and a specificity of 72.5%. 16

A meta-analysis of perioperative outcomes in open versus laparoscopic repair of perforated ulcers reported that there was no significant difference in terms of clinical outcome including mortality. 17 Therefore, lack of laparoscopic approach in the present study may have a limited impact in the overall study parameters.

A drawback of this study is that Graham's omental patch repair was done in all cases irrespective of the size of the perforation. However, study conducted by Chan et al has shown that the rate of intra-abdominal collection, reoperation, leak, and mortality was similar when either omental patch repair or gastrectomy was performed in giant PULPs. 18

With the above considerations, the present study requires further validation on a larger population to derive any conclusive results.

## Conclusion

The incidence and mortality in PPUs remain to be high. The present study conducted on 45 patients compared four scoring systems: the PULP score, the MPI, the ASA score, and the JS. To predict the mortality, the MPI and the JS were found to be better predictors with a significant *p* -value. The PULP score, as compared with the previous studies, did not perform well in predicting the mortality. The individual factors which are considered in formulating the score make it quite restrictive to a specific subgroup of patients with PULPs. This could be attributed to the difference in the demographics of this study population when compared with the other studies. Though the AUC for PULP score was found to be comparable to other previous studies, in the subset of Indian population, the JS would be much better suited as the variables involved are the most basic ones which can be made available even at a grass root level of health care.
